# Aminoglycosylation Can Enhance the G-Quadruplex Binding Activity of Epigallocatechin

**DOI:** 10.1371/journal.pone.0053962

**Published:** 2013-01-15

**Authors:** Li-Ping Bai, Hing-Man Ho, Dik-Lung Ma, Hui Yang, Wai-Chung Fu, Zhi-Hong Jiang

**Affiliations:** 1 State Key Laboratory of Quality Research in Chinese Medicine, and Macau Institute for Applied Research in Medicine and Health, Macau University of Science and Technology, Taipa, Macau; 2 School of Chinese Medicine, Hong Kong Baptist University, Kowloon Tong, Kowloon, Hong Kong; 3 Department of Chemistry, Hong Kong Baptist University, Kowloon Tong, Kowloon, Hong Kong; Florida International University, United States of America

## Abstract

With the aim of enhancing G-quadruplex binding activity, two new glucosaminosides (**16**, **18**) of penta-methylated epigallocatechin were synthesized by chemical glycosylation. Subsequent ESI-TOF-MS analysis demonstrated that these two glucosaminoside derivatives exhibit much stronger binding activity to human telomeric DNA and RNA G-quadruplexes than their parent structure (i.e., methylated EGC) (**14**) as well as natural epigallocatechin (EGC, **6**). The DNA G-quadruplex binding activity of **16** and **18** is even more potent than strong G-quadruplex binder quercetin, which has a more planar structure. These two synthetic compounds also showed a higher binding strength to human telomeric RNA G-quadruplex than its DNA counterpart. Analysis of the structure-activity relationship revealed that the more basic compound, **16**, has a higher binding capacity with DNA and RNA G-quadruplexes than its N-acetyl derivative, **18**, suggesting the importance of the basicity of the aminoglycoside for G-quadruplex binding activity. Molecular docking simulation predicted that the aromatic ring of **16** π-stacks with the aromatic ring of guanine nucleotides, with the glucosamine moiety residing in the groove of G-quadruplex. This research indicates that glycosylation of natural products with aminosugar can significantly enhance their G-quadruplex binding activities, thus is an effective way to generate small molecules targeting G-quadruplexes in nucleic acids. In addition, this is the first report that green tea catechin can bind to nucleic acid G-quadruplex structures.

## Introduction

Nucleic acid G-quadruplexes, four-stranded helical structures held together by a core of guanine tetrads, are secondary structures formed in particular G-rich sequences. Potential nucleic acid G-quadruplex structures have been identified in telomeric DNA and RNA sequences [1]–[5] as well as non-telomeric chromosomal promoters [6]–[9] of biological significance. These higher-order structures in nucleic acids represent a new class of molecular targets for selective DNA- and RNA-interacting compounds; in view of the fact that cancer cells have high telomerase activity and abnormal overexpression of oncogenes relative to normal cells, they are promising targets for cancer drug discovery [10]. In addition, numerous compounds have been designed to inhibit telomerase or to inactivate the transcription of oncogenes, such as *c-Myc*, *c-kit*, and *Bcl-2* [6], [9], [11]–[13], suggesting that the design of drugs targeting telomere or promoter G-quadruplexes is a rational and promising approach for generating new anticancer agents [14]. While recognition of G-quadruplex has mostly been achieved with the use of planar aromatic ligands through stacking interactions with the G-tetrad [14], grooves and negatively charged phosphate residues in G-quadruplexes are alternative binding sites to consider in the design of G-quadruplex stabilizing ligands [15]–[22].

Green tea catechins, the main biologically-active constituents of green tea, have gained significant recognition as cancer preventive agents. Green tea catechins are composed of four major polyphenols: (−)-epigallocatechin gallate (EGCG), (−)-epigallocatechin (EGC), (−)-epicatechin gallate (ECG), and (−)-epicatechin (EC) [23]. They show a variety of pharmacological activities, including cancer-preventive, antioxidant, anti-cancer [24], anti-angiogenesis activities [25], as well as inhibiting the fibrillogenesis of amyloid *β* peptide [26]–[27], anti-mutagenic and anti-viral activities [28]–[31]. It has been reported that catechins affect DNA replication, DNA repair, and transcription [32]–[34]. A recent study revealed that nucleic acids are binding targets of green tea catechins: nucleic acids extracted from EGCG-treated human cancer cells were catechin-colored, and direct binding of catechins with single-stranded and double-stranded DNA/RNA was observed by cold spray ionization-mass spectrometry [34]. However, a molecular docking study indicated that catechins, including EGC, are poor DNA G-quadruplex-stabilizing ligands compared with the more planar compound quercetin [35]. Therefore, structural modification of EGC is necessary for enhancing its G-quadruplex binding affinity.

It has been discovered that aminosugar moieties play an essential role in both the *in vitro* and *in vivo* antitumor activity of anthracyclines based on a DNA-binding mechanism [36]. It was also found that the N-acetyl glucosamine moiety seems to enhance the cytotoxic activity of the saponin julibroside III towards KB cancer cells [37]. A recent study found that non-planar aminoglycosides, such as neomycin and paromomycin, recognize the wide groove of *Oxytricha nova* telomeric G-quadruplex DNA [15]. These findings led to a prediction that the coupling of aminosugars with ligands that bind to G-quadruplex through stacking interactions may lead to enhanced G-quadruplex stabilizing properties. In our previous study, it was demonstrated that glycosylation of shikonin/alkannin with N-acetyl glucosamine is an effective way to generate a potent G-quadruplex DNA ligand [38]. Based on these observations, in this study we herein designed and synthesized two new glucosaminosides of EGC (**16**, **18**) and subsequently examined their binding affinities with both telomeric DNA and RNA G-quadruplexes by ESI-TOF-MS. Furthermore, the binding of these two glucosaminosides (**16**, **18**) with oncogene G-quadruplexes was also explored. Finally, the binding mode of **16** with human telomeric DNA G-quadruplex was investigated by computational docking experiments.

## Results

### Synthesis of EGC Glucosaminosides

**Figure 1:**
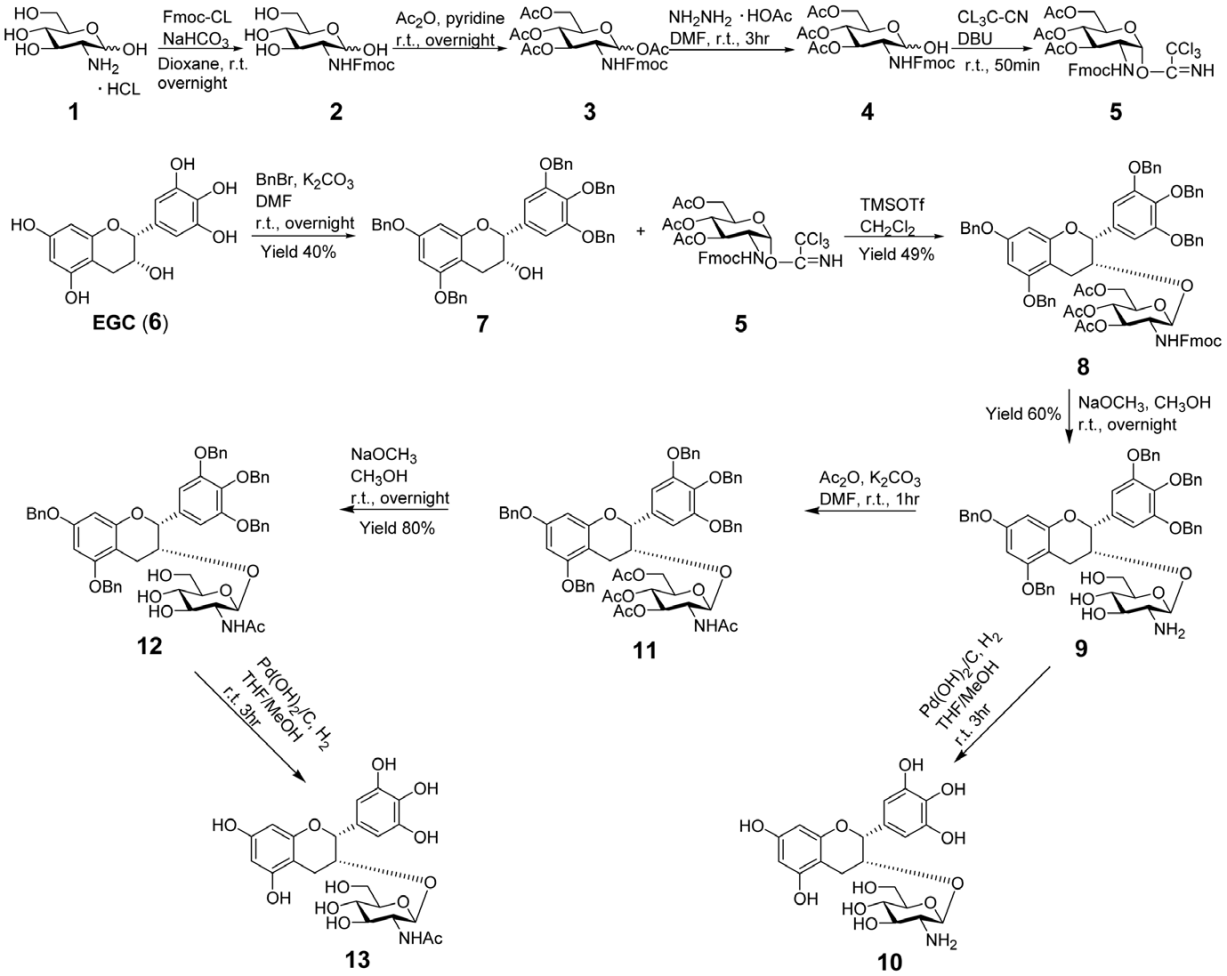
Figure **1. Synthesis of glucosaminosides of EGC.**

Glycosylation is an effective method for connecting saccharide units to natural products in order to obtain biologically active glycosides [39]–[40]. Many glycosylated natural products have been reported to show high activity against a variety of human tumors [36]–[37]. In this study, chemical glycosylation was employed as a key approach to acquiring EGC glucosaminosides. As illustrated in Figure 1, our initial efforts were focused on the design and synthesis of EGC-3-*O*-*β*-glucosaminoside (**10**) and its N-acetyl derivative (**13**), starting from the readily available (−)-EGC and D-(+)-glucosamine hydrochloride. The glycosyl donor **5** was prepared according to the method previously described in the literature [38], [41]–[42]. The amino group of glucosamine was firstly blocked by 9-fluorenylmethoxycarbonyl chloride (Fmoc-Cl) and followed by acetylation of the hydroxyl groups. After selective deprotection, the anomeric hydroxyl group was transformed to trichloroacetimidate and thus activated for glycosylation [38].Trimethylsilyl triflate (TMSOTf) was used as a catalyst for the glycosylation of the penta-benzyl ether of EGC (**7**). After deprotection and acetylation of the amino group, EGC glucosaminoside [**10**, ESI-TOF-MS *m/z* (C_21_H_26_NO_11_)^+^: calcd 468.1500, found 468.1489] and its N-acetyl product **13** [ESI-TOF-MS *m/z* (C_23_H_28_NO_12_)^+^: calcd 510.1606, found 510.1599] were synthesized. Unfortunately, we failed to purify these two products due to their instability during the course of column chromatographic purification.

**Figure 2:**
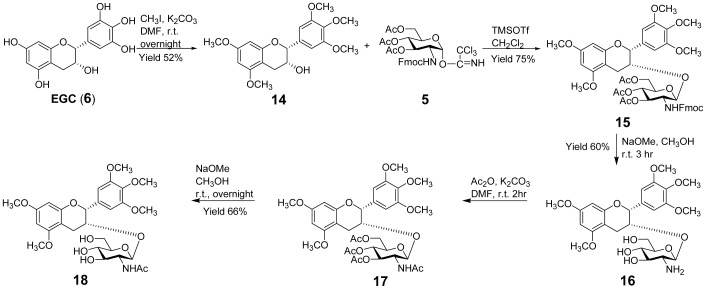
Synthesis of glucosaminosides of penta-methylated EGC.

To avoid this instability, we slightly modified our target products by blocking the active phenolic hydroxyl groups of EGC with methyl groups. The penta-methylated EGC (**14**) was subsequently glycosylated with glycosyl donor **5** by the action of TMSOTf, followed by deprotection to give **16**. Its N-acetyl derivative **18** was synthesized by further acetylation of **16** and subsequent deacetylation (Figure 2). Compounds **16** and **18** were characterized to be 5, 7, 3′, 4′, 5′-penta-*O*-methyl epigallocatechin *β*-D-glucosaminoside and 5, 7, 3′, 4′, 5′-penta-*O*-methyl epigallocatechin N-acetyl *β*-D-glucosaminoside, respectively, on the basis of ^1^H-NMR, ^13^C-NMR and high resolution mass spectroscopic evidence (Figure S1, S2, S3, S4, S5, S6, S7, S8, S9) [43]–[44].

### Analysis of Human Telomeric G-quadruplex DNA Binding by ESI-TOF-MS

Mass spectrometry coupled with the source of electrospray ionization (ESI), a soft ionization method, has played a more active role in the investigation of noncovalent complexes of nucleic acids with small organic molecules. It has the advantages of direct assignment of the stoichiometry and gives an indication of the relative amounts of different species of complexes [45]–[46]. Mass spectrometry, combined with techniques of ion mobility and molecular dynamics, has demonstrated that DNA G-quadruplexes in telomeric repeats are conserved in a solvent-free environment [47].

The G-quadruplex DNA-binding activities of glucosaminosides of penta-methylated EGC (**16**, **18**), as well as their aglycone (**14**) and natural EGC (**6**), were examined with a 27 nt human telomeric sequence d[(TTAGGG)_4_TTA] which forms an intramolecular G-quadruplex, by ESI-TOF-MS. Quercetin, a flavonoid with a similar but more planar structure than EGC, was used as a reference compound for the comparison of G-quadruplex binding activity of the natural and synthetic compounds, since it was reported to be stacked with terminal tetrads of monomeric G-quadruplexes [48].

**Figure 3:**
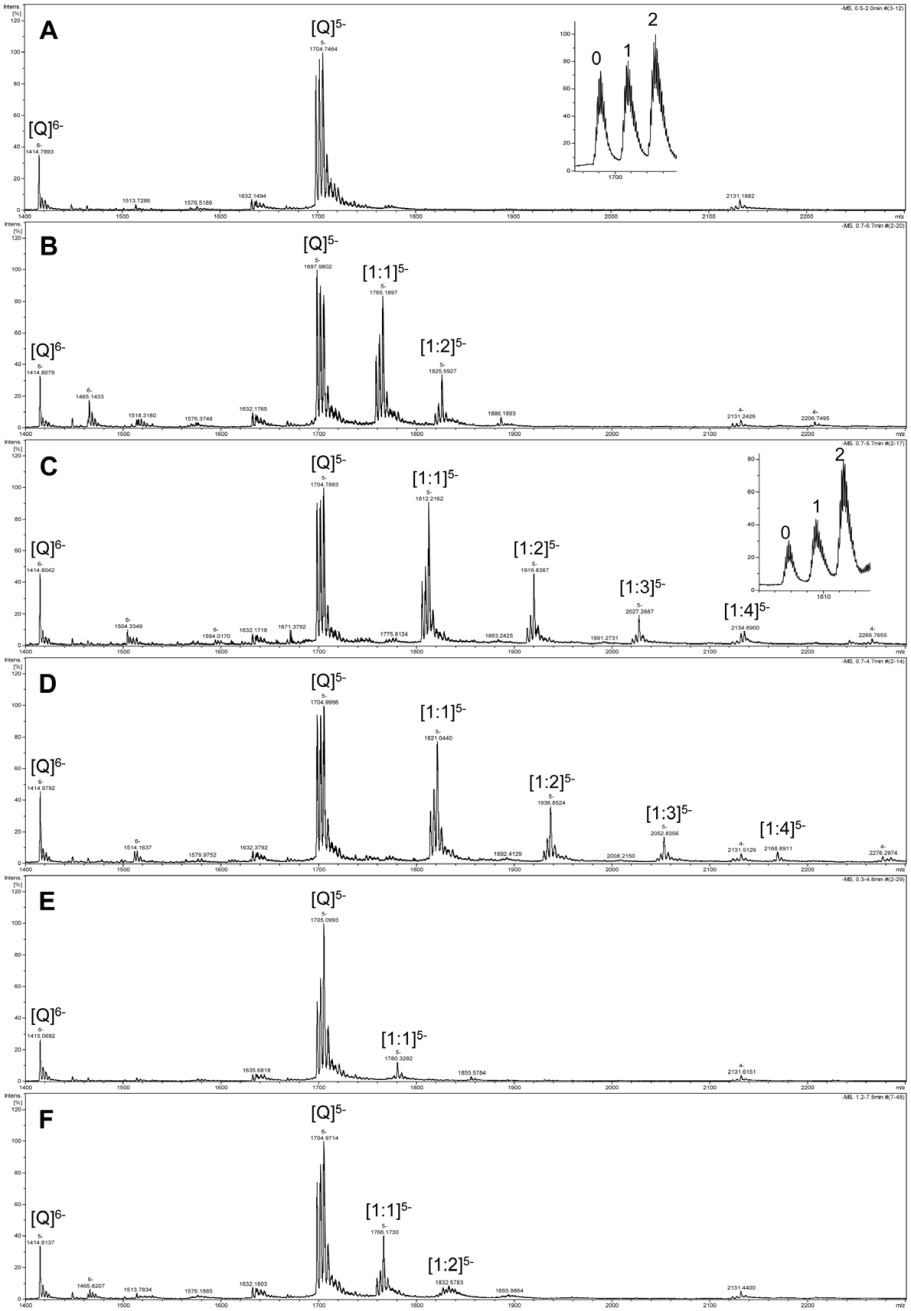
ESI-TOF-MS spectra of telomeric DNA d[(TTAGGG)_4_TTA] (Q) in the absence and presence of drugs. Negative ESI-TOF-MS spectra of human telomeric DNA sequence d[(TTAGGG)_4_TTA] were recorded under conditions of (A) without drug, (B) with quercetin, (C) with compound **16**, (D) with compound **18**, (E) with compound **14** and (F) with EGC. The inserts in spectra A and C are the enlargements of free G-quadruplex and complex ions with 1∶1 binding stoichiometry, respectively. The numbers in the inserts represent the number of ammonium ion adducts. Spectra were recorded with a molar ratio of DNA/drug of 1∶1 (C = 50 µM) in 50 mM ammonium acetate buffer (pH 7.6) containing 50% methanol.

**Figure 4:**
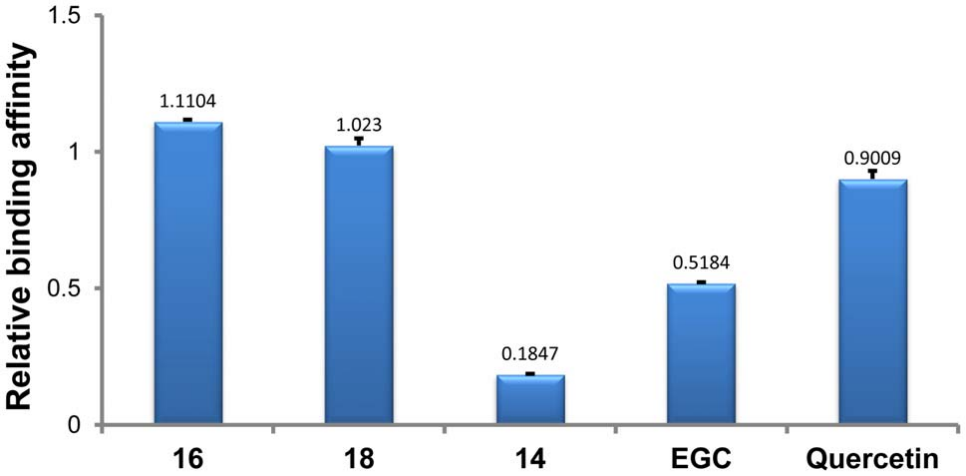
Relative binding affinities of drugs with intramolecular human telomeric DNA G-quadruplex. The numbers indicated on the top of each column showed the mean values from two determinations.

The ESI-TOF-MS spectrum of telomeric DNA showed that the addition of the NH_4_OAc buffer facilitated the detection of quadruplex (Q^5−^ in Figure 3A) [49] in the −5 charge state ions at *m/z* 1697.9, 1701.3, and 1704.7. These three ions correspond to the lone oligodeoxynucleotide and the oligodeoxynucleotides with one and two NH_4_^+^ ion adducts, respectively. When the drug was added to DNA, the complex peaks with two NH_4_^+^ ion adducts became more predominant than those with one NH_4_^+^ ion adduct or none when a molar ratio of DNA/drug of 1∶1 was used (Figure 3B–F). This indicated that the G-quadruplex structure stabilized by drugs holds NH_4_^+^ ions inserted between G-tetrads more tightly than free G-quadruplex in the course of being introduced into the gas phase. In other words, drug-bound G-quadruplex is more stable than when it is unbound. In order to compare the stabilization effect of different molecules on DNA G-quadruplex, the peak area ratio of all [complex]^5−^ to [quadruplex]^5−^ was used to evaluate the relative binding affinities (Figure 4) [49]–[51].

As illustrated in Figure 4, the relative binding affinities of all tested drugs with intramolecular human telomeric DNA G-quadruplex followed the descending order of **16**>**18**> Quercetin>EGC >**14**. Two synthetic glucosaminosides (**16** and **18**) demonstrated a more strong stabilizing effect on intramolecular human telomeric DNA G-quadruplex than their parent structure, methylated EGC (**14**). These two glucosaminosides also showed higher relative binding affinities than the natural catechin EGC and the even more planar flavonol quercetin. This indicated that the introduction of a glucosamine moiety into penta-methylated EGC (**14**), the weakest G-quadruplex binder among all tested compounds, resulted in a largely enhanced G-quadruplex stabilizing ability. This finding was further supported by the results of UV-melting study [15]–[22] that the melting temperature (*T*_m_) of dAGGG(TTAGGG)_3_ was increased 3.92, 1.86, 0.34 and 0°C?by the presence of 50 µM of **16**, **18**, EGC and **14**, respectively. On the basis of the above results, the following structure-activity relationships can be summarized. First, the more basic compound **16** demonstrated more potent G-quadruplex DNA-binding capacity than compound **18**, suggesting the importance of basicity of the aminoglycoside in G-quadruplex DNA-binding activity. Secondly, the distinct binding behaviors of EGC and its penta-methylated derivative (**14**) to G-quadruplex DNA indicated that the hydroxyls in EGC are essential groups for its G-quadruplex DNA-binding activity.

It was also found that both a glucosaminoside of methylated EGC (**16**) and its acetyl-N derivative (**18**) bind to the intramolecular human telomeric DNA G-quadruplex with 1∶1, 1∶2, 1∶3 and 1∶4 stoichiometries when a molar ratio of DNA/drug of 1∶1 is used. However, their aglycone **14** only shows 1∶1 binding stoichiometry under the same condition. The multiple stoichiometries of **16** and **18** binding with intramolecular human telomeric DNA G-quadruplex suggested that, unlike their aglycone, these two glucosaminosides of penta-methylated EGC bind to multiple sites on the human telomeric DNA G-quadruplex.

### Molecular Modeling of Methylated EGC Glucosaminoside Derivatives Binding with Human Telomeric DNA G-quadruplex

**Figure 5:**
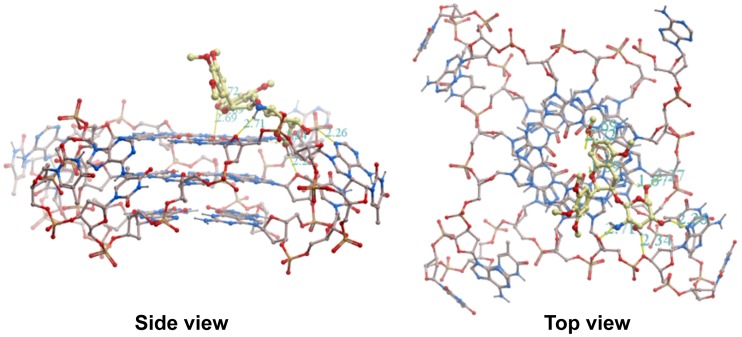
Molecular modeling of 16 binding with the human intramolecular telomeric G-quadruplex (PDB code: 1KF1). Oxygen atoms are highlighted in red, nitrogen atoms in blue, phosphorus atoms in yellow and carbon atoms in beige.

Using **16** as a model compound, a molecular modeling study was performed on its binding with intramolecular human telomeric G-quadruplex (PDB code: 1KF1 [52]) to provide insight into the binding mode of aminoglucosides of methylated EGC. As illustrated in Figure 5, the aglycone moiety of **16** is predicted to bind to the 5′ terminal face of the G-quadruplex through stacking interactions between the aromatic rings of methylated EGC and guanine nucleotides. The part of the glucosamine moiety of **16** is predicted to reside in the grooves of the G-quadruplex through hydrogen bonding interactions between donors in the G-quadruplex and hydrogens in both the amino and hydroxyl groups of **16**. The amino hydrogen of **16** forms a hydrogen bond with the oxygen atom of the deoxyribose in the phosphate backbone. The hydroxyl hydrogens of **16** form hydrogen bonds with oxygen atoms from the phosphate sugars and adenine residues of the G-quadruplex. This kind of binding mode, towards the top of the 5′ terminus of the G-quadruplex, is the most favorable binding interaction, with a binding energy of −35.99 kJ/mol.

### Analysis of Binding with Oncogene G-quadruplex DNA by ESI-TOF-MS

**Figure 6:**
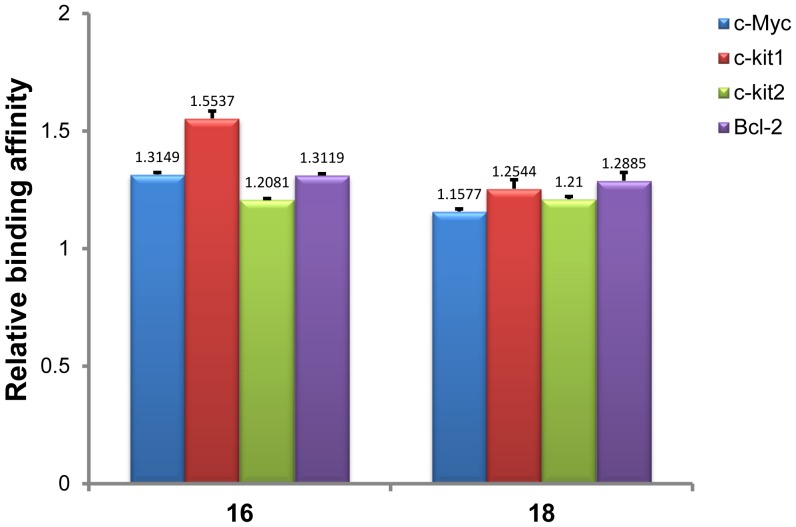
Relative binding affinities of 16 and 18 with different intramolecular oncogene G-quadruplexes. The experiments were conducted with a molar ratio of DNA/drug of 1∶1 (100 µM:100 µM) in 100 mM ammonium acetate (pH 7.6) containing 50% methanol. The numbers indicated on the top of each column showed the mean values from two determinations.

In addition to human telomeric G-quadruplex DNA, the binding of two synthetic glucosaminosides of methylated EGC (**16**, **18**) with oncogene G-quadruplexes derived from the sequences of c-Myc, c-kit1, c-kit2 and Bcl-2 was further investigated by the same ESI-TOF-MS technique. All the oncogene sequences displayed the ability to form G-quadruplex structures in NH_4_OAc buffer, as ESI-TOF-MS spectra revealed that the major ion in −5 charge state of each sequence corresponds to the *m/z* value of the oligodeoxynucleotide with two NH_4_^+^ ions adduct (Figure S10). As with telomeric DNA G-quadruplex, **16** and **18** also showed multiple binding stoichiometries with all oncogene DNA G-quadruplexes. The relative binding affinities presented in Figure 6 show that **16** demonstrated comparative binding strength with c-Myc, c-kit1, c-kit2, and Bcl-2 DNA G-quadruplexes. Compound **18** exhibited almost equivalent binding capacity with all oncogene G-quadruplexes. These results demonstrate that glucosaminosides (**16**, **18**) of methylated EGC exhibit binding capacity to different oncogene G-quadruplexes, though without obvious G-quadruplex selectivity.

### Analysis of Interaction with Human Telomeric RNA G-quadruplex by ESI-TOF-MS

**Figure 7:**
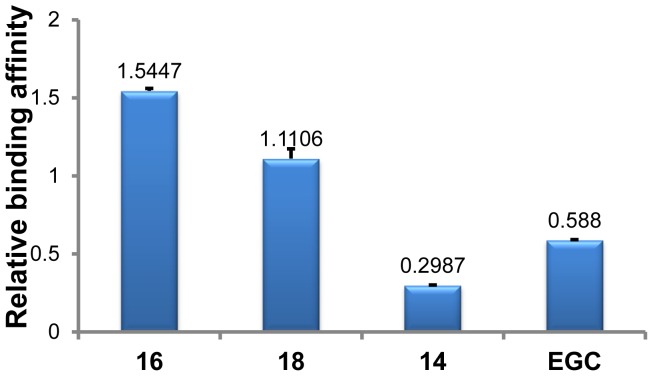
Relative binding affinities of drugs with intramolecular human telomeric RNA G-quadruplex. The numbers indicated on the top of each column showed the mean values from two determinations.

**Figure 8:**
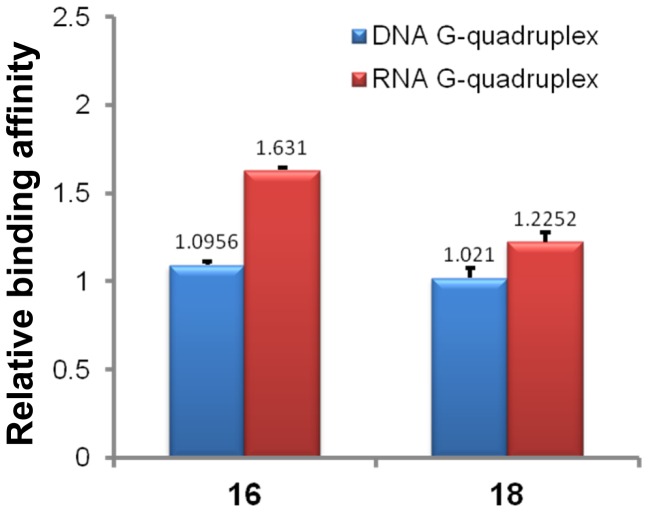
Relative binding affinities of drugs with DNA and RNA G-quadruplex in competitive binding experiments. The numbers indicated on the top of each column showed the mean values from two determinations.

A recent finding demonstrated that telomere DNA is transcribed into telomeric repeat-containing RNA in mammalian cells. The telomeric repeat-containing RNA sequence, r(UUAGGG)_4_, folds into a parallel G-quadruplex in solution that is more stable than its DNA counterpart [53]–[56]. The binding of synthetic glucosaminosides (**16**, **18**) of penta-methylated EGC, penta-methylated EGC (**14**) and EGC (**6**) were therefore studied with a 27 nt human telomeric RNA sequence, r[(UUAGGG)_4_UUA], under the same ESI-TOF-MS conditions as used for its DNA counterpart (Figure S11) [55]–[56]. As shown in Figure 7, the RNA G-quadruplex binding strength of these four compounds also followed the same descending trend of **16**>**18**> EGC >**14** as seen with a DNA G-quadruplex. By Comparing with the DNA G-quadruplex binding results (Figure 4), it was found that the relative affinity of each compound for the RNA G-quadruplex was slightly higher. In order to confirm this result, competitive binding experiments were carried out for two methylated EGC glucosaminosides (**16**, **18**) with both DNA and RNA G-quadruplexes to further elucidate the DNA and RNA G-quadruplex binding selectivity of each compound (Figure S12). In each competition experiment, human telomeric DNA and RNA G-quadruplexes were mixed with each compound to give a final molar ratio of DNA/RNA/compound of 1∶1∶2. The peak area ratio of drug-bound complex to free nucleic acid of each species of G-quadruplex was calculated to give the value of relative affinity (Figure 8), which confirmed that both **16** and **18** show stronger binding affinity with RNA G-quadruplex than DNA G-quadruplex. To provide further evidence to support the ESI-TOF-MS results of the above binding studies of these aminoglucosaminosides, we undertook UV-melting experiments [15]–[22]. It was demonstrated that **16** and **18** increased melting temperature of rAGGG(UUAGGG)_3_ 6.08 and 3.26°C, respectively, under the same tested condition with that of DNA analogue. The larger Δ*T*_m_ value for RNA G-quadruplex further confirmed that **16** and **18** exhibited stronger stabilizing effects on RNA G-quadruplex than DNA counterpart. However, there are no obvious increases for the melting temperature of a double-stranded oligodeoxynucleotide (ds-DNA) 5′-AGGGTTAGGGT-3′/3′-TCCCAATCCCA-5′ in the presence of **16** (0.22°C)?and **18** (−0.15°C), indicating the selectivity of two synthetic compounds to RNA and DNA G-quadruplexex over duplex oligonucleotide (Figure S13).

## Discussion and Conclusion

In this study, two glucosaminosides of methylated EGC, compounds **16** and **18,** have been successfully synthesized for the first time. Both the DNA and RNA G-quadruplex binding activity of these two glucosaminosides has been evaluated and compared with that of green tea catechin EGC by ESI-TOF-MS analysis. The DNA and RNA G-quadruplex binding capacity of both synthetic compounds and natural EGC with human telomeric DNA and RNA sequences both followed the order of **16**>**18**> EGC >>**14**. This finding indicated that introduction of a glucosamine moiety to penta-methylated EGC (**14**), the weakest G-quadruplex binder among all tested compounds, resulted in much stronger G-quadruplex stabilizing ability that exceeds natural EGC. In addition, they exhibited stronger DNA G-quadruplex binding activity than the more planar structure quercetin. Analysis of the structure-activity relationship revealed that the more basic glucosaminoside **16** generally showed more potent G-quadruplex binding capacity than that of **18**, indicating the importance of basicity of aminoglycoside in G-quadruplex binding activity. Furthermore, it was demonstrated that both glucosaminosides of penta-methylated EGC had a greater binding affinity with the RNA G-quadruplex than its DNA counterpart. Additionally, it has been shown that glucosaminosides **16** and **18** can also bind to different oncogene G-quadruplexes, although without obvious G-quadruplex selectivity. Taken together, these results demonstrate that aminoglycosylation of natural products is an effective way to design and synthesize small molecules targeting G-quadruplexes in nucleic acids.

ESI-TOF-MS analysis revealed that glucosaminosides **16** and **18** demonstrated more binding stoichiometries with an intramolecular human telomeric G-quadruplex than EGC under a molar ratio of DNA/drug of 1∶1, suggesting that there are more binding sites for **16** and **18** in the intramolecular human telomeric G-quadruplex than the natural tea catechin EGC and their aglycone (**14**). Subsequent molecular docking simulation predicted that the aromatic ring of compound **16** π-stacked with the aromatic ring of guanine nucleotides, with the glucosamine moiety residing in the groove of G-quadruplex. This prediction is consistent with the binding mode of aminoglycosides neomycin and paramonomycin [15]. This kind of binding mode is also in agreement with the multiple binding stoichiometries of **16** with the 27 nt human telomeric G-quadruplex detected by ESI-TOF-MS.

Although green tea catechins were proven to bind to normal (single-stranded and double-stranded) DNA and RNA [28]–[31], this is the first time they were also found to bind to DNA and RNA G-quadruplex structures. The distinct binding behaviors of EGC and its penta-methylated derivative, compound **14**, to DNA and RNA G-quadruplexes suggested that the hydroxyl groups in EGC are essential for stabilizing DNA and RNA G-quadruplexes. The binding of nucleic acid G-quadruplexes by green tea catechins may be in part responsible for their cancer-preventive activities.

## Materials and Methods

### General

The trichloroacetimidate method was employed to conduct glycosylation reaction, which was performed in the presence of TMSOTf at −40°C under an atmosphere of argon, followed by deprotection of sugar moiety with sodium methoxide in methanol and further catalytic hydrogenation with palladium hydroxide on carbon powder. Unless otherwise noted, all reactions were conducted in oven-dried glassware. Column chromatography for product purification was performed on DAVISIL® chromatographic silica gel LC60A (40–63 micron, GRACE Davison, Germany), and Chromatorex ODS (100–200 mesh, Fuji Silysia Chemical Ltd., Japan). Purity checking of product was accomplished on an ultra performance LC system (Acquity™, Waters) equipped with photodiode array detector (Waters) and a Bruker micrOTOF ESI-TOF mass spectrometer by using an Acquity UPLC® BEH C_18_ column (17 µM, 2.1×100 mm, part No. 186002352, Ireland). ^1^H and ^13^C NMR were recorded at room temperature on a Bruker 400 MHz NMR Avance-III (Switzerland) or a Varian 400 MHz NMR Inova 400 (USA) spectrometers operating at 400 MHz (^1^H) and 100 MHz (^13^C). Coupling constants were given in Hz and chemical shifts were represented in δ (ppm) relative to Me_4_Si as internal standard. HR-ESI-MS was performed on a Q-TOF mass spectrometer (Bruker Daltonics, MA, U.S.A). Optical rotation was measured on a JASCO P-1010 polarimeter (Japan) with a 1 dm cell (C given in g/100 mL).

### 3,4,5-tri-*O*-acetyl-2-deoxy-2-(9-Fluorenylmethoxycarbonylamino)-α-D-glucopyranosyl Trichloroacetimidate (5)

To a solution of glucosamine chloride (8.15 g, 37.8 mmol) in water (50 mL), NaHCO_3_ (4.76 g, 56.7 mmol) and 9-fluorenylmethoxycarbonylchloride (11.7 g, 45.4 mmol) in dioxane (50 mL) were added and the reaction stirred at room temperature until silica gel TLC (CHCL_3_-EtOAc-MeOH 7∶2∶3) showed complete conversion of the starting material to N-protected glucosamine (**2**). The reaction solution was slowly poured into ice water (900 mL) and filtered under decreased pressure after stirring at room temperature for 2 h. The residue was washed by water and dried at 50°C to afford crude **2** (13.8 g). The N-protected glucosamine (**2**, 13.8 g, 34.6 mmol) in pyridine (50 mL) was acetylated with acetic anhydride (18 mL, 173 mmol) by stirring overnight at room temperature to produce tetra-*O*-acetyl carbohydrate (**3**). The reaction solution was poured into ice water and filtered under decreased pressure, followed by silica gel column chromatography (PE-acetone 7∶3) to give **3** (18.5 g). The anomeric acetyl group of **3** (8.65 g, 15.2 mmol) in DMF (50 mL) was then deprotected with hydrazine acetate (1.54 g, 16.72 mmol) by stirring at room temperature for 5 h. The reaction solution was then diluted with EtOAc (300 mL), then washed with water (100 mL×4). The organic layer was dried with anhydrous Na_2_SO_4_ and concentrated to give **4** (7.3 g)**.** To a solution of **4** (1.844 g, 3.50 mmol) in anhydrous CH_2_Cl_2_ (60 mL), CCl_3_-CN (2.91 mL, 13.97 mmol) and DBU (58.2 µL, 0.35 mmol) was added. After stirring at room temperature for 50 minutes, the reaction mixture was directly loaded to silica gel chromatography column eluted with n-hexane-EtOAc (6∶4) containing 0.5% triethylamine to afford **5** (2.25 g, 96% yield).

### (−)-5, 7, 3′, 4′, 5′-Penta-*O*-methyl Epigallocatechin (14)

To a solution of epigallocatechin (612 mg, 2.0 mmol) in DMF (20 mL), methyl iodide (1.0 mL, 16.0 mmol) and K_2_CO_3_ (2.21 g, 16.0 mmol) was added. The mixture was stirred at room temperature for 15 h. 180 mL of water was poured into the reaction solution, followed by extraction with ethyl acetate (100 mL × 4). The organic solutions was dried with anhydrous Na_2_SO_4_ and subjected to silica gel chromatography (n-hexane-ethyl acetate 9∶1 to 5∶5) to afford **14** (388 mg, 52% yield) as a white amorphous powder. [*α*]_D_^20^ −76.16 (C = 0.44, EtOAc). HRMS (ESI) *m/z* (C_20_H_25_O_7_)^+^: calcd 377.1595, found 377.1583. ^1^H-NMR (400 MHz, CDCl_3_) *δ*: 4.94 (s, H-2), 4.30 (m, H-3), 2.98 (dd, *J* = 1.6, 17.3 Hz, H-4β), 2.90 (dd, *J* = 4.3, 17.3 Hz, H-4*α*), 6.21 (d, *J* = 2.3, H-6), 6.13 (d, *J* = 2.3 Hz, H-8), 6.75 (2H, s, H-2′, 6′), 3.86 (3H, s, 4′-OCH_3_), 3.80, 3.78 (3H each, s, 5, 7-OCH_3_), 3.90 (6H, s, 3′, 5′-OCH_3_), 1.78 (d, *J* = 5.6 Hz, 3-OH). ^13^C-NMR (100 MHz, DMSO) *δ*: 78.4 (C-2), 64.4 (C-3), 28.6 (C-4), 101.0 (C-4a), 158.9, 158.7 (C-5, C-7), 93.4 (C-6), 91.4 (C-8), 155.4 (C-8a), 135.2 (C-1′), 104.4 (C-2′, C-6′), 152.5 (C-3′, C-5′), 136.7 (C-4′), 55.4, 55.2 (5, 7-OCH_3_), 55.9 (3′, 5′-OCH_3_), 60.0 (4′-OCH_3_).

### Compound 15

A heterogeneous mixture of compound **14** (113 mg, 0.30 mmol) and trichloroacetimidate **5** (336 mg, 0.50 mmol) in anhydrous CH_2_Cl_2_ (6 mL) with 4 Å molecular sieves (500 mg) was stirred at room temperature for 30 min under an argon atmosphere and then was cooled to −40°C. TMSOTf (600 µL, 0.06 M in dry CH_2_CL_2_) was added quickly to the precooled reaction mixture and the resulting mixture was allowed to warm to room temperature over 3 h. Triethylamine (24 µL) was added to quench the reaction. The organic solution was then directly loaded to silica gel chromatograph column eluted with n-hexane-ethyl acetate (from 9∶1 to 5∶5) to give **15** (199.1 mg, 75% yield). HRMS (ESI) *m/z* (C_47_H_52_NO_16_)^+^: calcd 886.3281, found 886.3275.

### (−)-5, 7, 3′, 4′, 5′-penta-*O*-methyl Epigallocatechin *β*-D-glucosaminoside (16)

Compound **15** (177 mg, 0.20 mmol) was dissolved in methanol (14 mL) and sodium methoxide (40 mg, 0.74 mmol) was added. After 3 h of stirring at room temperature, the reaction solution was filtered and evaporated under reduced pressure. The residue was purified by silica gel chromatography eluted with chloroform-methanol-water (from 90∶10∶1 to 80∶20∶2) to afford **16** (64 mg, 60% yield) as a white amorphous powder. Optical rotation [*α*]_D_^20^ −30.48 (C = 0.44, MeOH). HRMS (ESI) *m/z* (C_26_H_36_NO_11_)^+^: calcd 538.2283, found 538.2271. ^1^H-NMR (400 MHz, DMSO and D_2_O) *δ*: 5.14 (s, H-2), 4.50 (m, H-3), 2.72 (dd, *J* = 3.6, 17.4 Hz, H-4a), 2.67 (dd, *J* = 4.0, 17.4 Hz, H-4b), 6.12 (d, *J* = 2.3 Hz, H-6), 6.09 (d, *J* = 2.3 Hz, H-8), 6.84 (2H, s, H-2′, 6′), 3.71, 3.65 (3H each, s, 5, 7-OCH_3_), 3.72 (6H, s, 3′, 5′-OCH_3_), 3.74 (3H, s, 4′-OCH_3_), 4.26 (d, *J* = 7.8 Hz, glc-1), 2.31 (dd, *J* = 8.0, 9.0 Hz, glc-2), 3.04 (t, *J* = 9.0 Hz, glc-3), 2.96 (t, *J* = 9.0 Hz, glc-4), 3.12 (m, glc-5), 3.68 (m, overlapped with H_2_O peak, glc-6a), 3.34 (dd, *J* = 6.4, 11.6 Hz, glc-6b). ^13^C-NMR (100 MHz, DMSO) *δ*: 77.5 (C-2), 70.1 (C-3), 23.9 (C-4), 101.3 (C-4a), 159.6, 158.9 (C-5, 7), 93.6 (C-6), 91.9 (C-8), 155.6 (C-8a), 134.2 (C-1′), 105.6 (C-2′, 6′), 152.6 (C-3′, 5′), 137.1 (C-4′), 100.9 (glc-1), 57.5 (glc-2), 76.4 (glc-3), 70.6 (glc-4), 77.4 (glc-5), 61.6 (glc-6), 55.9, 55.6 (5, 7-OCH_3_), 56.2 (3′, 5′-OCH_3_), 60.5 (4′-OCH_3_).

### Compound 17

To a solution of compound **16** (16 mg, 0.03 mmol) in DMF (2 mL), acetic anhydride (40 µL, 0.39 mmol) and K_2_CO_3_ (12 mg, 0.09 mmol) was added at room temperature. After 2 hours of stirring, ice water (20 mL) was poured into the mixture solution to stop reaction. Then ethyl acetate (10 mL × 3) was used to extract the target product. The organic solution was dried with anhydrous Na_2_SO_4_ and evaporated to give **17** (15.6 mg, 74% yiled). HRMS (ESI) *m/z* (C_34_H_44_NO_15_)^+^: calcd 706.2705, found 706.2695.

### (−) 5, 7, 3′, 4′, 5′-penta-*O*-methyl Epigallocatechin N-acetyl *β*-D-glucosaminoside (18)

Sodium methoxide (6.06 mg, 0.11 mmol) was added to a solution of **17** (15.6 mg, 0.022 mmol) in methanol (3 mL). The mixture was allowed to stir at room temperature overnight. After neutralization by formic acid (100 µL), the reaction solution was loaded to an ODS column eluted with 70% methanol to give **18** (8.5 mg, 66% yield) as a white amorphous powder. [*α*]_D_^20^ −47.12 (C = 0.42, MeOH). HRMS (ESI) *m/z* (C_28_H_38_NO_12_)^+^: calcd 580.2389, found 580.2377. ^1^H-NMR (400 MHz, CD_3_OD) *δ*: 5.04 (s, H-2), 4.49 (m, H-3), 2.92 (dd, *J* = 3.6, 17.3 Hz, H-4a), 2.70 (dd, *J* = 3.9, 17.3 Hz, H-4b), 6.13 (d, *J* = 2.3 Hz, H-6), 6.10 (d, *J* = 2.3 Hz, H-8), 6.91 (2H, s, H-2′, 6′), 3.77, 3.74 (3H each, s, 5, 7-OCH_3_), 3.85 (6H, s, 3′, 5′-OCH_3_), 3.80 (3H, s, 4′-OCH_3_), 4.58 (d, *J* = 7.8 Hz, glc-1), 3.82 (m, glc-2), 3.45–3.58 (3H, m, glc-6a, 6b, 3), 3.21–3.24 (2H, m, glc-4, 5), 1.58 (3H, s, -COCH_3_). ^13^C-NMR (100 MHz, CD_3_OD) *δ*: 79.7 (C-2), 71.9 (C-3), 24.6 (C-4), 101.8 (C-4a), 161.2, 160.5 (C-5, 7), 94.6 (C-6), 92.4 (C-8), 157.3 (C-8a), 136.1 (C-1′), 106.8 (C-2′, 6′), 153.9 (C-3′, 5′), 138.5 (C-4′), 99.7 (glc-1), 57.7 (glc-2), 75.6 (glc-3), 72.5 (glc-4), 78.0 (glc-5), 63.1 (glc-6), 56.1, 56.0 (5, 7-OCH_3_), 56.9 (3′, 5′-OCH_3_), 61.2 (4′-OCH_3_), 173.7 (C = O in acetyl group), 22.7 (CH_3_ in acetyl group).

### Mass Spectrometry

All ESI-MS experiments were carried out on a Bruker MicrOTOFQ mass spectrometer in negative ion mode, with the capillary voltage set to +3500 V, the dry N_2_ gas flow set to 4.0 L/min at 100 celcius, and injection flow rate of sample set to 3 µL/min. Data processing was performed by the software Bruker Daltonics DataAnalysis. All the nucleic acids oligomers (desalted grade) were purchased from Invitrogen and used without further purification. The stock solutions of all nucleic acid oligomers were prepared in milli Q water at the concentration of 1 mM, and further diluted by 1 M NH_4_OAc buffer (pH 7.6) to the desired concentration. All stock solutions of drugs were prepared in methanol at a concentration of 400 µM. For the analysis of the noncovalent complex of oncogene DNA G-quadruplex with drug, the samples were prepared at a final concentration of 100 µM DNA and 100 µM drug in 100 mM NH_4_OAc (pH 7.6) containing 50% methanol. For the analysis of human telomeric DNA and RNA G-quadruplexes, the samples were injected at a final strand concentration of 50 µM oligomer and 50 µM drug in 50 mM NH_4_OAc (pH 7.6) containing 50% methanol. Each sample of nucleic acid-drug complex solution was prepared in duplicate.

The oligonucleotide sequences are shown as the following:

- human telomeric DNA: 5′-TTAGGGTTAGGGTTAGGGTTAGGGTTA-3′ (*M* = 8496.6241 Da)
- c-*myc* DNA: 5′-TGGGGAGGGTGGGGAGGGTGGGGAAGG-3′ (*M* = 8687.7137 Da)
- c-*kit* 1 DNA: 5′-AGAGGGAGGGCGCTGGGAGGAGGGGCT-3′ (*M* = 8576.6545 Da)
- c-*kit* 2 DNA: 5′-CCCGGGCGGGCGCGAGGGAGGGGAGGT-3′ (*M* = 8513.5935 Da)
- truncated *bcl*-2 DNA: 5′-CGGGCGCGGGAGGAAGGGGGCGGGAGC-3′ (*M* = 8562.6312 Da)
- human telomeric RNA: 5′-UUAGGGUUAGGGUUAGGGUUAGGGUUA-3′ (*M* = 8789.3336 Da)

### Molecular Modeling

A computer model to study the binding of **16** with human telomeric DNA G-quadruplex was performed by using the literature method [57]. Molecular modeling was performed using the ICM-Pro 3.4-8a program (Molsoft). The X-ray crystal structure of the intramolecular G-quadruplex DNA was obtained from the Protein Data Bank (PDB code: 1KF1) and used as the model to perform molecular modeling [57].

### UV-melting Study

UV-melting profiles were recorded by using a Beckman Coulter DU800® spectrophotometer equipped with a high performance temperature controller. The absorbance was monitored at 295 nm for G-quadruplex in 25 mM Tris-HCl buffer (pH 7.0) containing 5 mM KCl and 1% DMSO, and at 260 nm for duplex oligonucleotides in 25 mM Tris-HCl buffer (pH 7.0) containing 1% DMSO. The concentration of all oligonucleotides was 5 µM for the Δ*T*_m_ measurement in the absence and presence of compounds (50 µM).

## Supporting Information

Figure S1 **Positive ESI-TOF-MS spectrum of compound 14.**
(TIF)

Figure S2 **^1^H-NMR spectrum of compound 14.** The spectrum was taken in CDCl_3_ at room temperature.
(TIF)

Figure S3 **^13^C-NMR spectrum of compound 14.** The spectrum was taken in DMSO-*d*6 at room temperature.
(TIF)

Figure S4 **Positive ESI-TOF-MS spectrum of compound 16.**
(TIF)

Figure S5 **^1^H-NMR spectrum of compound 16.** The spectrum was taken in the mixture of DMSO and D_2_O at room temperature.
(TIF)

Figure S6 **^13^C-NMR spectrum of compound 16.** The spectrum was taken in the mixture of DMSO and D_2_O at room temperature.
(TIF)

Figure S7 **Positive ESI-TOF-MS spectrum of compound 18.**
(TIF)

Figure S8 **^1^H-NMR spectrum of compound 18.** The spectrum was taken in CD_3_OD at room temperature.
(TIF)

Figure S9 **^13^C-NMR spectrum of compound 18.** The spectrum was taken in CD_3_OD at room temperature.
(TIF)

Figure S10 **Negative ESI-TOF-MS spectra of oncogene G-rich sequences.** (A–B) c-Myc sequence d[(TG_4_AG_3_)_2_TG_4_A_2_G_2_] with compound **16** and compound **18**. (C–D) c-kit1 sequence d[AG(AG_3_)_2_CGCTG_3_AG_2_AG_4_CT] with compound **16** and compound **18**. (E–F) c-kit2 sequence d[C_3_G_3_CG_3_(CG)_2_AG_3_AG_4_AG_2_T] with compound **16** and compound **18**. (G–H) truncated Bcl-2 sequence d[CG_3_CGCG_3_AG_2_A_2_G_5_CG_3_AGC] with compound **16** and compound **18**. Q represents quadruplex oligodeoxynucleotides. Spectra were recorded with 1∶1 DNA-to-drug molar ratio (C = 100 µM) in 50 mM ammonium acetate buffer (pH 7.6) containing 50% methanol.
(TIF)

Figure S11 **Negative ESI-TOF-MS spectra of human telomeric RNA sequence r[(UUAGGG)_4_UUA] (Q).** (A) without drug, (B) with compound **16**, (C) with compound **18**, (D) with compound **14,** and (E) with EGC. Spectra were recorded with 1∶1 DNA-to-drug molar ratio (C = 50 µM) in 50 mM ammonium acetate buffer (pH 7.6) containing 50% methanol.
(TIF)

Figure S12 **ESI-TOF-MS spectra of human telomeric DNA and RNA G-quadruplex in competitive binding experiments.** (A) an equal molar mixture of human telomeric DNA d[(TTAGGG)_4_TTA] (Q) and RNA r[(UUAGGG)_4_UUA] (Q) without drug, (B) with compound **16**, and (C) with compound **18**. Spectra were recorded with 1∶1∶2 molar ratio of DNA:RNA:drug (25 µM: 25 µM: 50 µM) in 50 mM ammonium acetate buffer (pH 7.6) containing 50% methanol.
(TIF)

Figure S13 **Thermal denaturation profiles of oligonucleotides in the absence and presence of 16.** (A) UV-melting profiles of rAGGG(UUAGGG)_3_ (5 µM) in the absence and presence (50 µM) of compound **16** in 25 mM Tris-HCl buffer (pH 7.0) containing 5 mM KCl and 1% DMSO, (B) UV-melting profiles of dAGGG(TTAGGG)_3_ (5 µM) in the absence and presence (50 µM) of compound **16** in 25 mM Tris-HCl buffer (pH 7.0) containing 5 mM KCl and 1% DMSO, (C) UV-melting profiles of double-stranded oligodeoxynucleotide (Ds-DNA) 5′-AGGGTTAGGGT-3′/3′-TCCCAATCCCA-5′ (5 µM) in the absence and presence (50 µM) of compound **16** in 25 mM Tris-HCl buffer (pH 7.0) containing 1% DMSO.
(TIF)
