# Supplementary figures and images for: Aminoglycosylation Can Enhance the G-Quadruplex Binding Activity of Epigallocatechin
Source: PLoS One. 2013 Jan 15;8(1):e53962. doi: 10.1371/journal.pone.0053962 (PMC3545880; doi:10.1371/journal.pone.0053962)

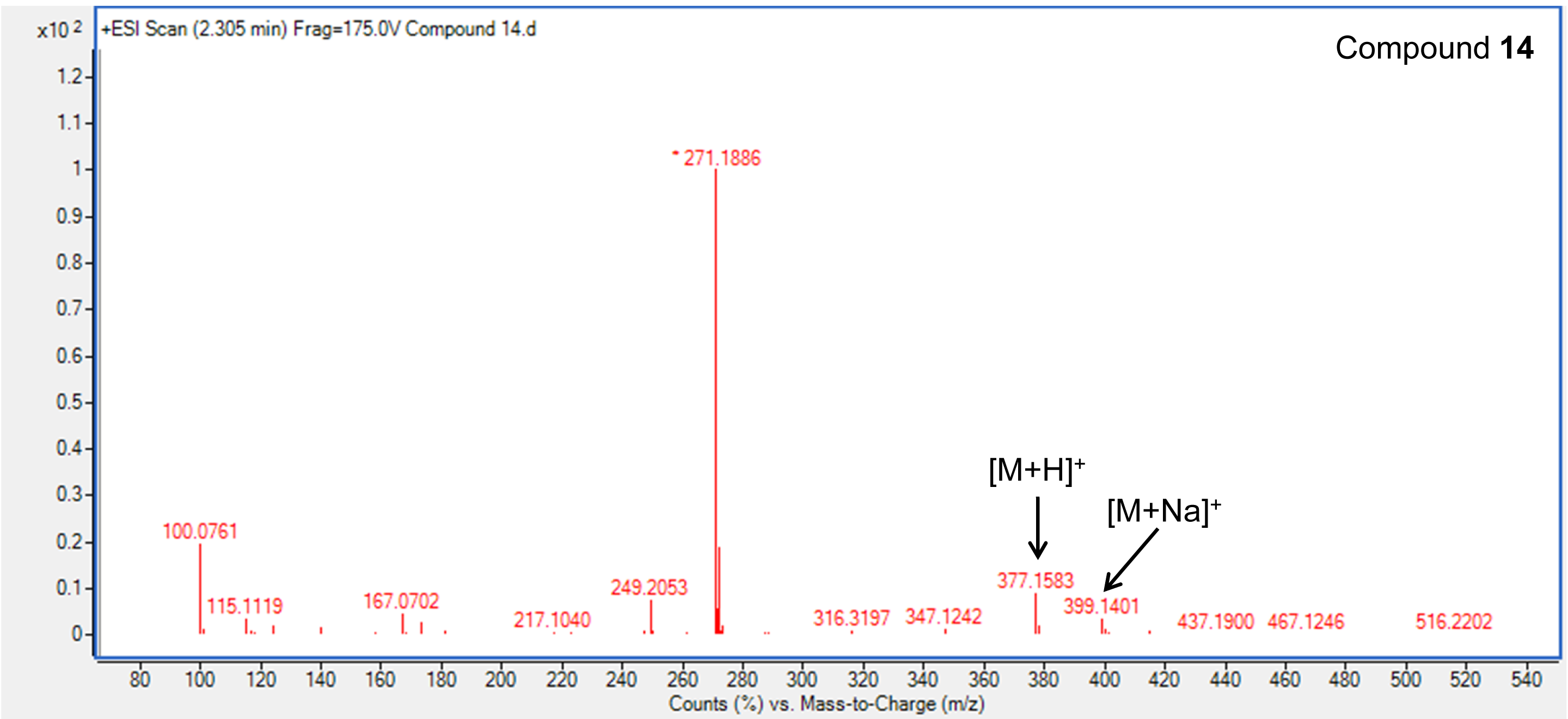

Supplement: Figure S1 — Positive ESI-TOF-MS spectrum of compound 14. (TIF) [file pone.0053962.s001.tif]

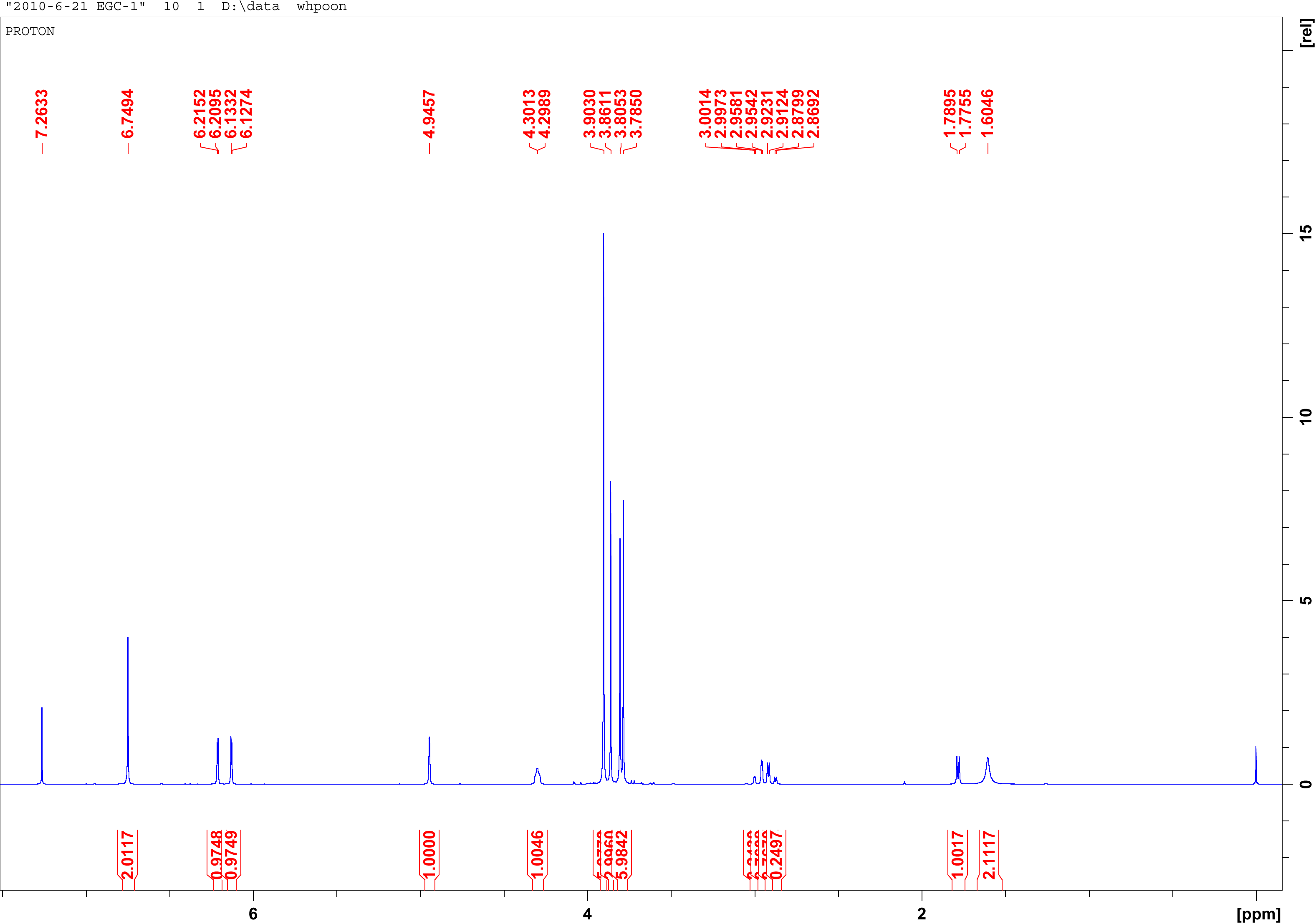

Supplement: Figure S2 — 1H-NMR spectrum of compound 14. The spectrum was taken in CDCl3 at room temperature. (TIF) [file pone.0053962.s002.tif]

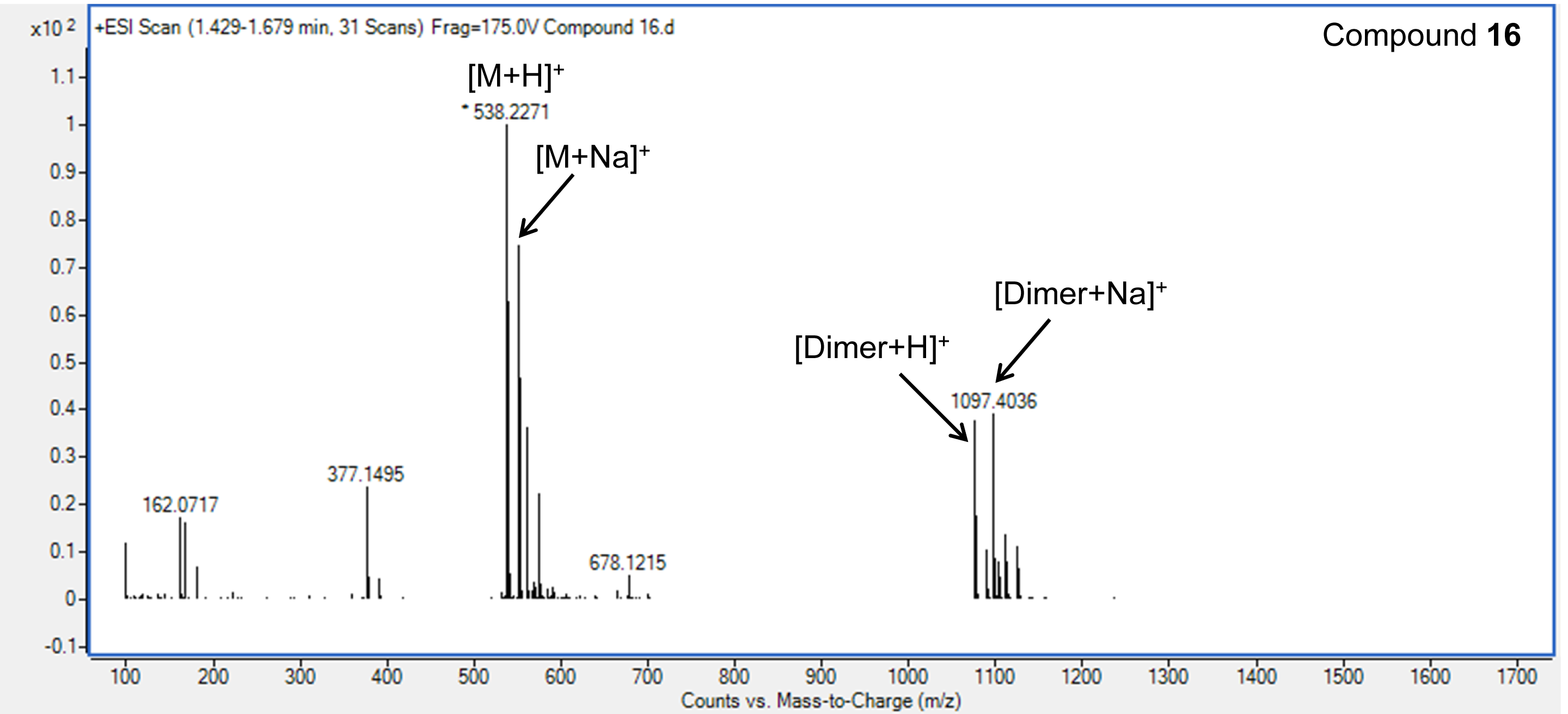

Supplement: Figure S4 — Positive ESI-TOF-MS spectrum of compound 16. (TIF) [file pone.0053962.s004.tif]

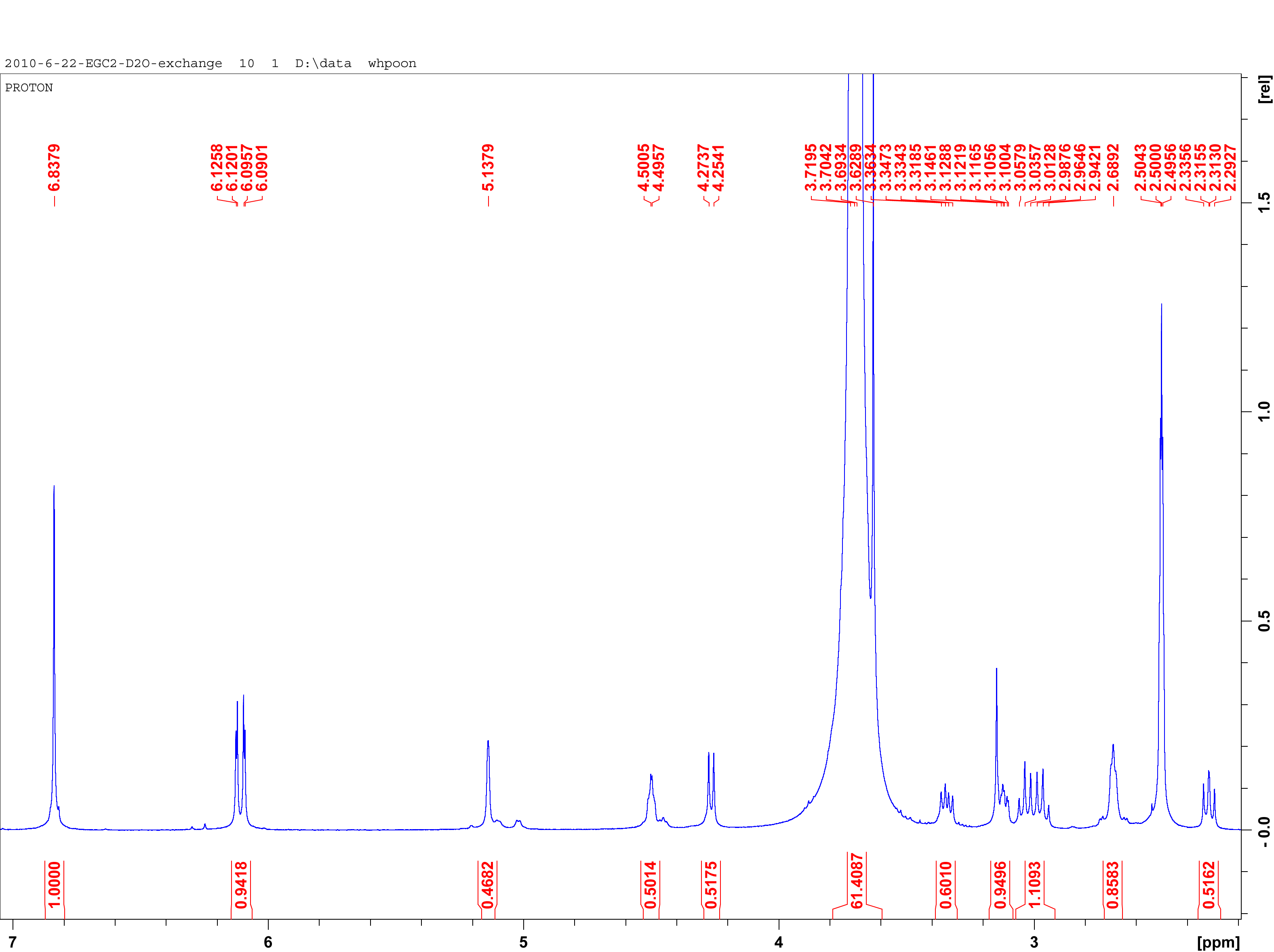

Supplement: Figure S5 — 1H-NMR spectrum of compound 16. The spectrum was taken in the mixture of DMSO and D2O at room temperature. (TIF) [file pone.0053962.s005.tif]

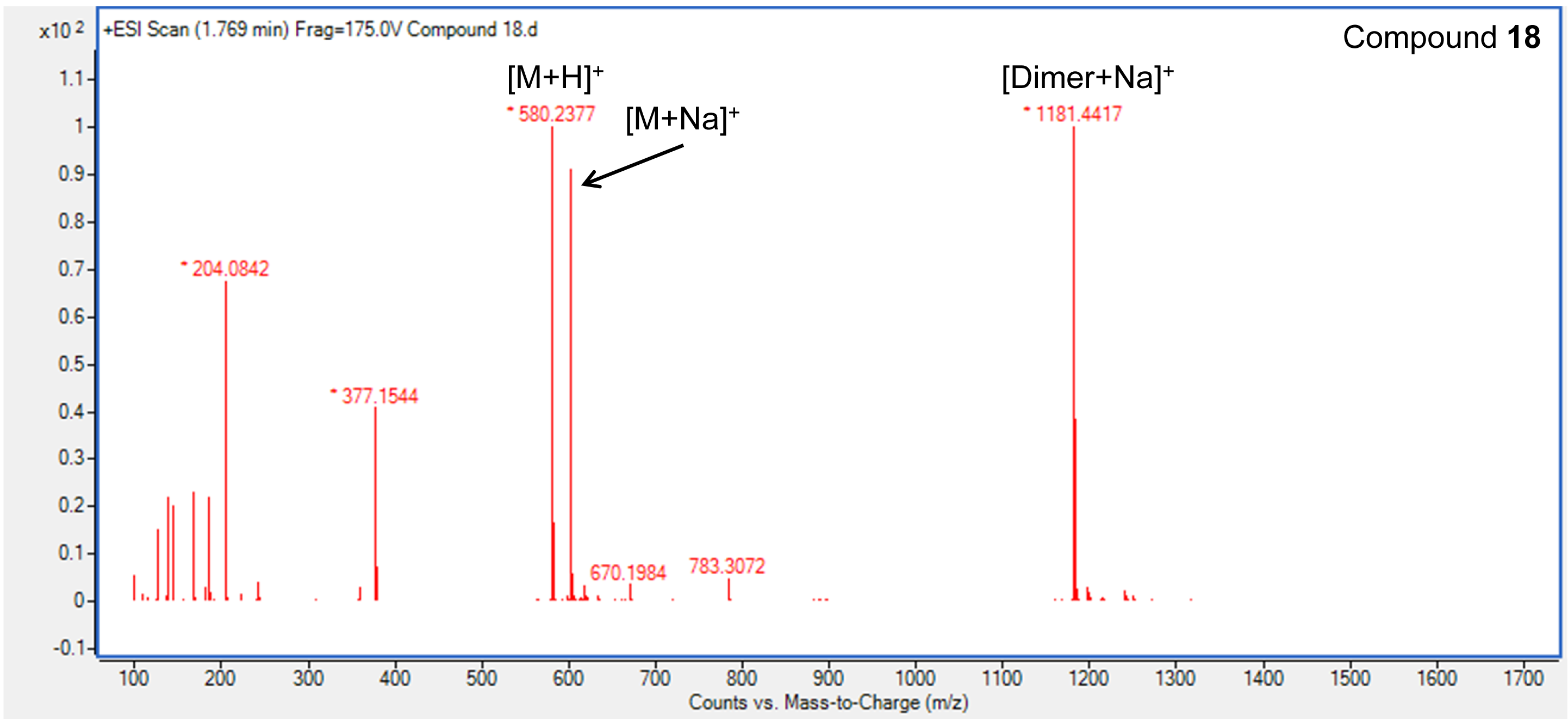

Supplement: Figure S7 — Positive ESI-TOF-MS spectrum of compound 18. (TIF) [file pone.0053962.s007.tif]

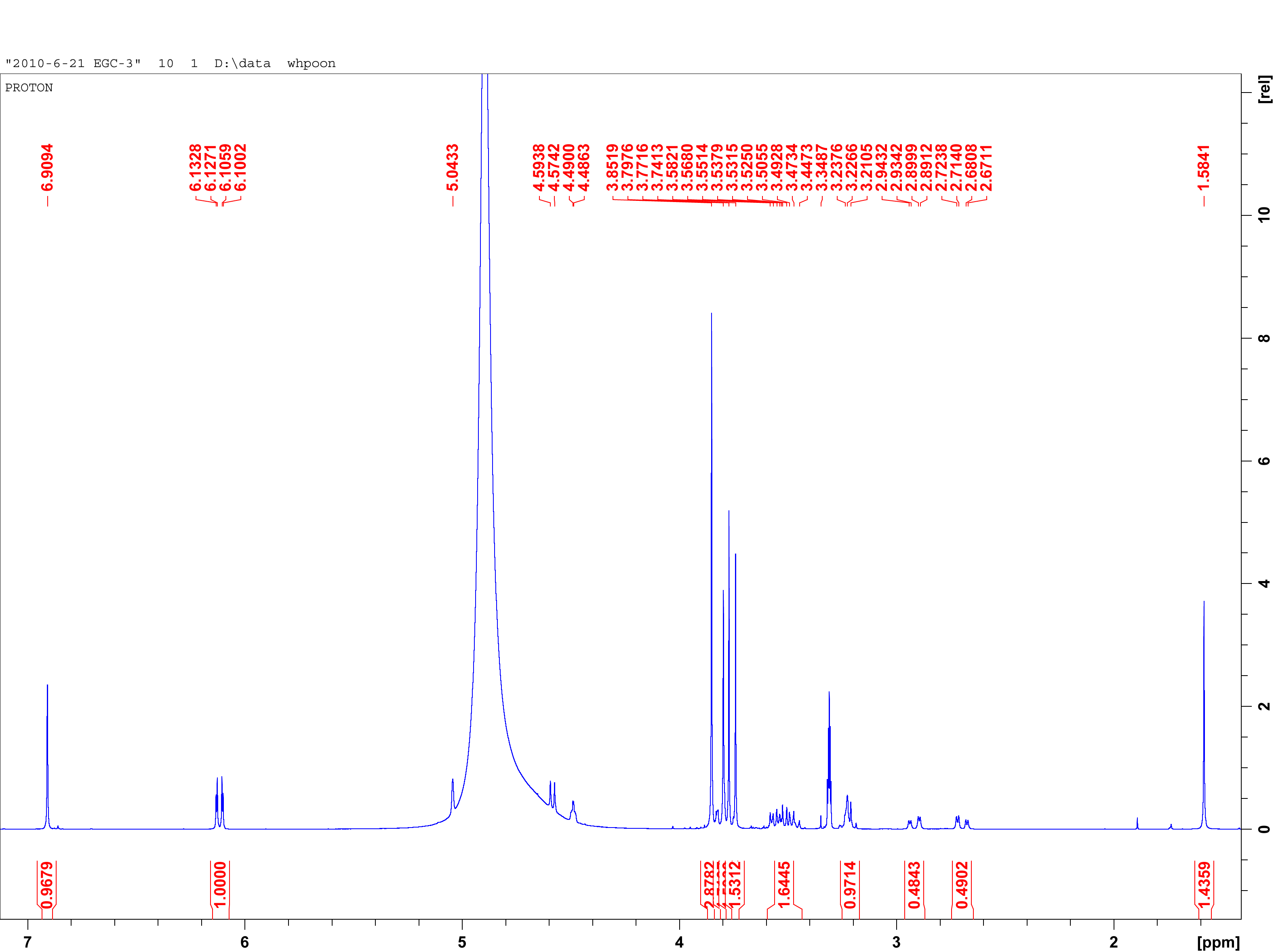

Supplement: Figure S8 — 1H-NMR spectrum of compound 18. The spectrum was taken in CD3OD at room temperature. (TIF) [file pone.0053962.s008.tif]

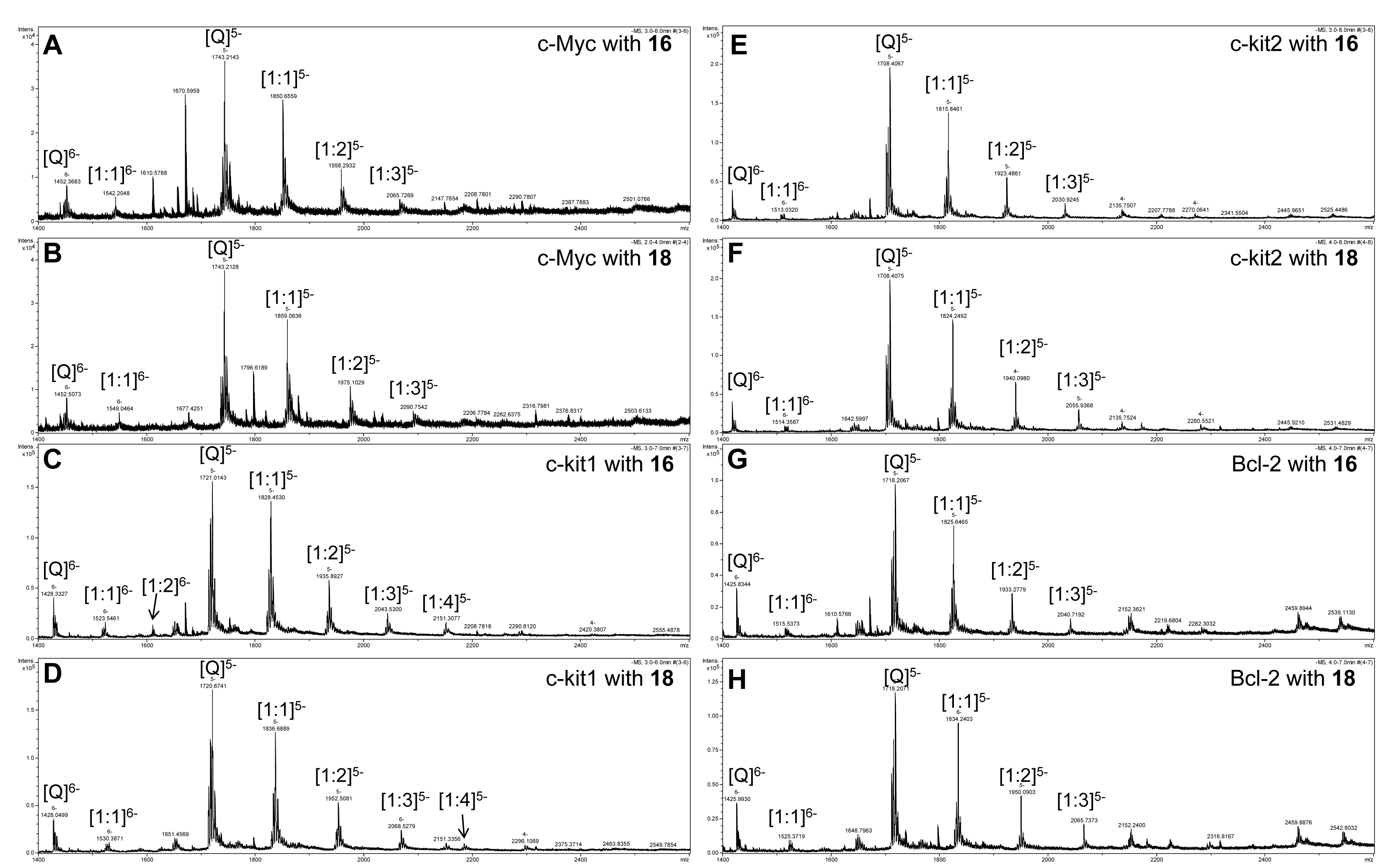

Supplement: Figure S10 — Negative ESI-TOF-MS spectra of oncogene G-rich sequences. (A–B) c-Myc sequence d[(TG4AG3)2TG4A2G2] with compound 16 and compound 18. (C–D) c-kit1 sequence d[AG(AG3)2CGCTG3AG2AG4CT] with compound 16 and compound 18. (E–F) c-kit2 sequence d[C3G3CG3(CG)2AG3AG4AG2T] with compound 16 and compound 18. (G–H) truncated Bcl-2 sequence d[CG3CGCG3AG2A2G5CG3AGC] with compound 16 and compound 18. Q represents quadruplex oligodeoxynucleotides. Spectra were recorded with 1∶1 DNA-to-drug molar ratio (C = 100 µM) in 50 mM ammonium acetate buffer (pH 7.6) containing 50% methanol. (TIF) [file pone.0053962.s010.tif]

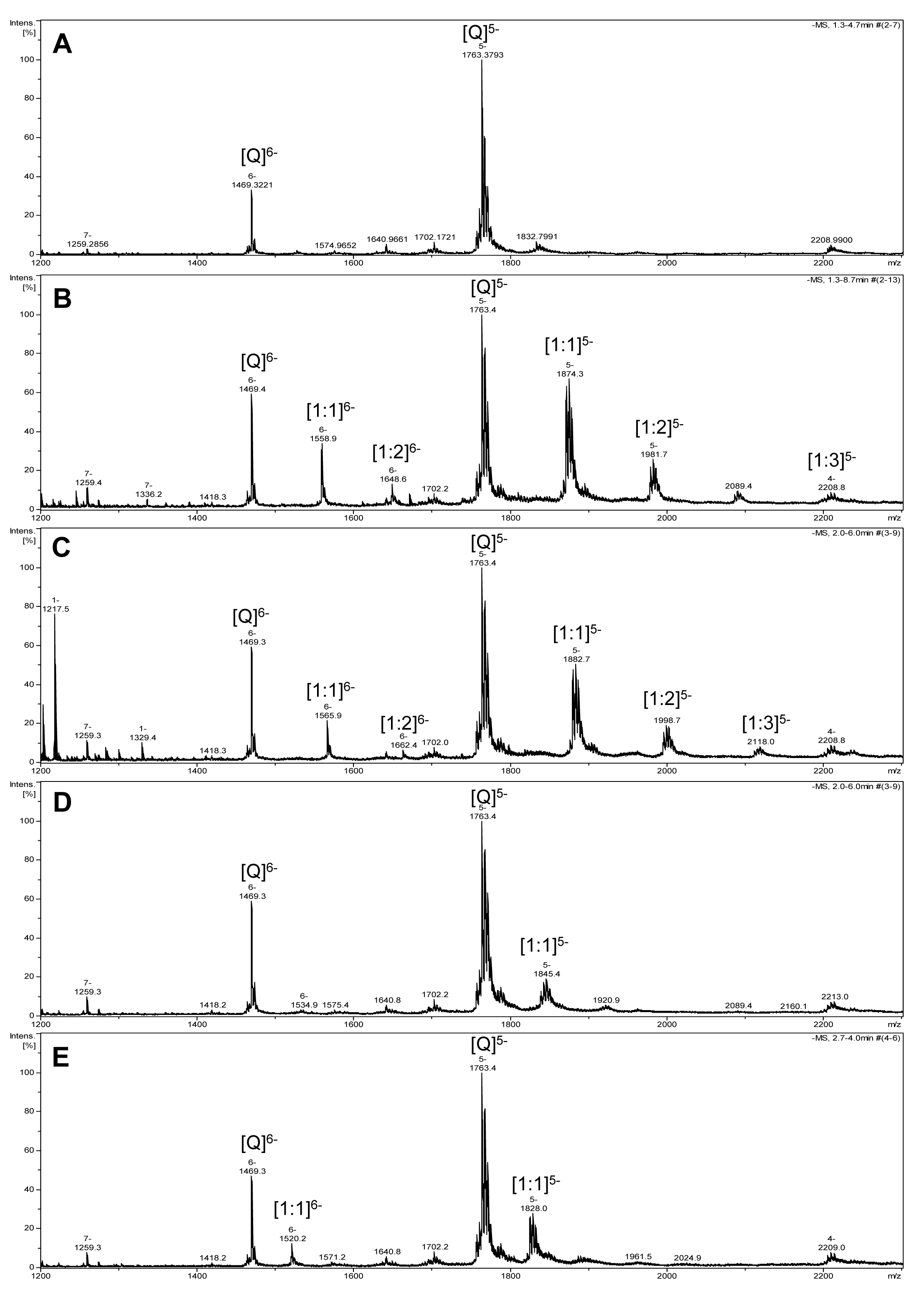

Supplement: Figure S11 — Negative ESI-TOF-MS spectra of human telomeric RNA sequence r[(UUAGGG)4UUA] (Q). (A) without drug, (B) with compound 16, (C) with compound 18, (D) with compound 14, and (E) with EGC. Spectra were recorded with 1∶1 DNA-to-drug molar ratio (C = 50 µM) in 50 mM ammonium acetate buffer (pH 7.6) containing 50% methanol. (TIF) [file pone.0053962.s011.tif]

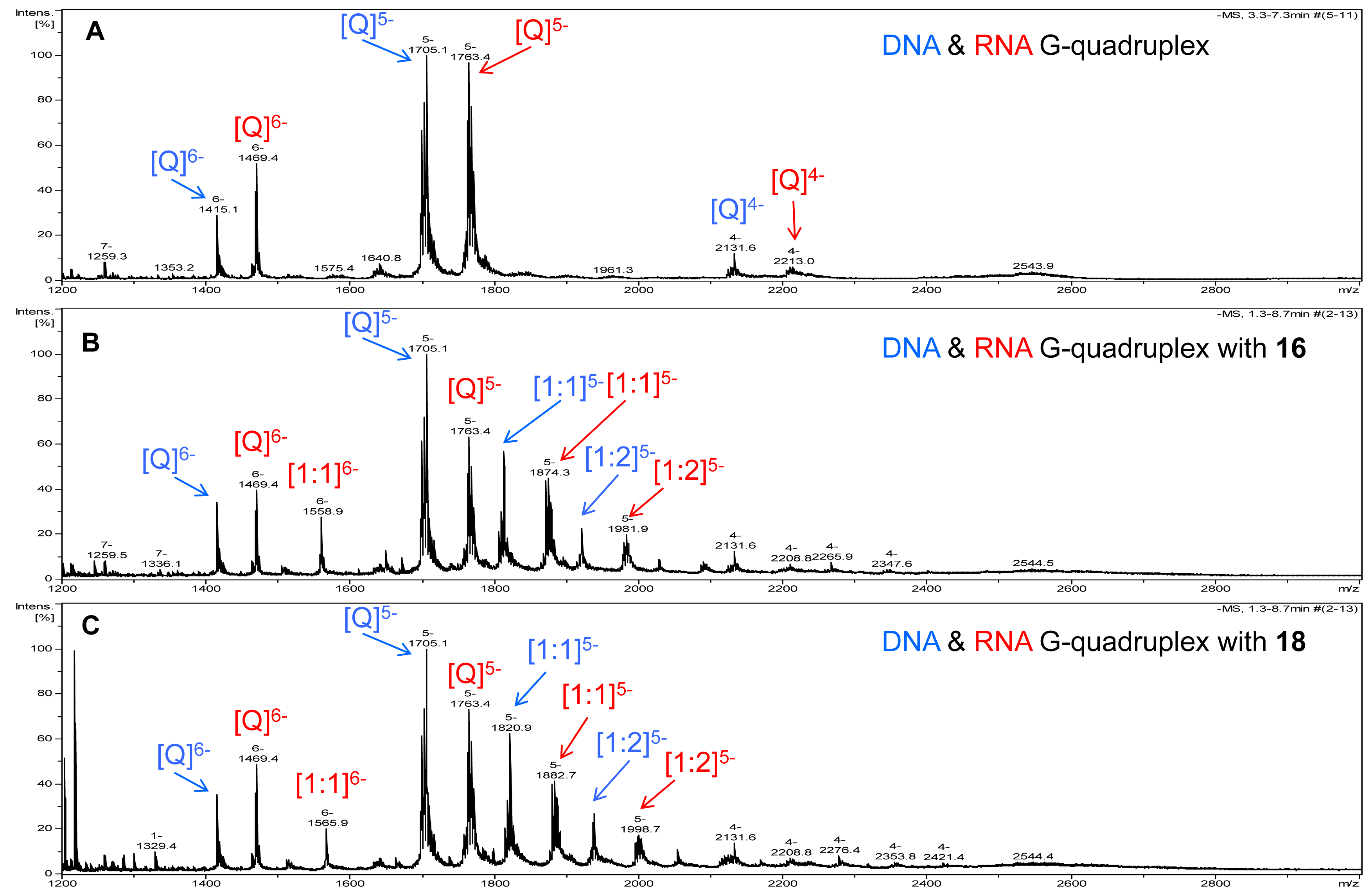

Supplement: Figure S12 — ESI-TOF-MS spectra of human telomeric DNA and RNA G-quadruplex in competitive binding experiments. (A) an equal molar mixture of human telomeric DNA d[(TTAGGG)4TTA] (Q) and RNA r[(UUAGGG)4UUA] (Q) without drug, (B) with compound 16, and (C) with compound 18. Spectra were recorded with 1∶1∶2 molar ratio of DNA:RNA:drug (25 µM: 25 µM: 50 µM) in 50 mM ammonium acetate buffer (pH 7.6) containing 50% methanol. (TIF) [file pone.0053962.s012.tif]

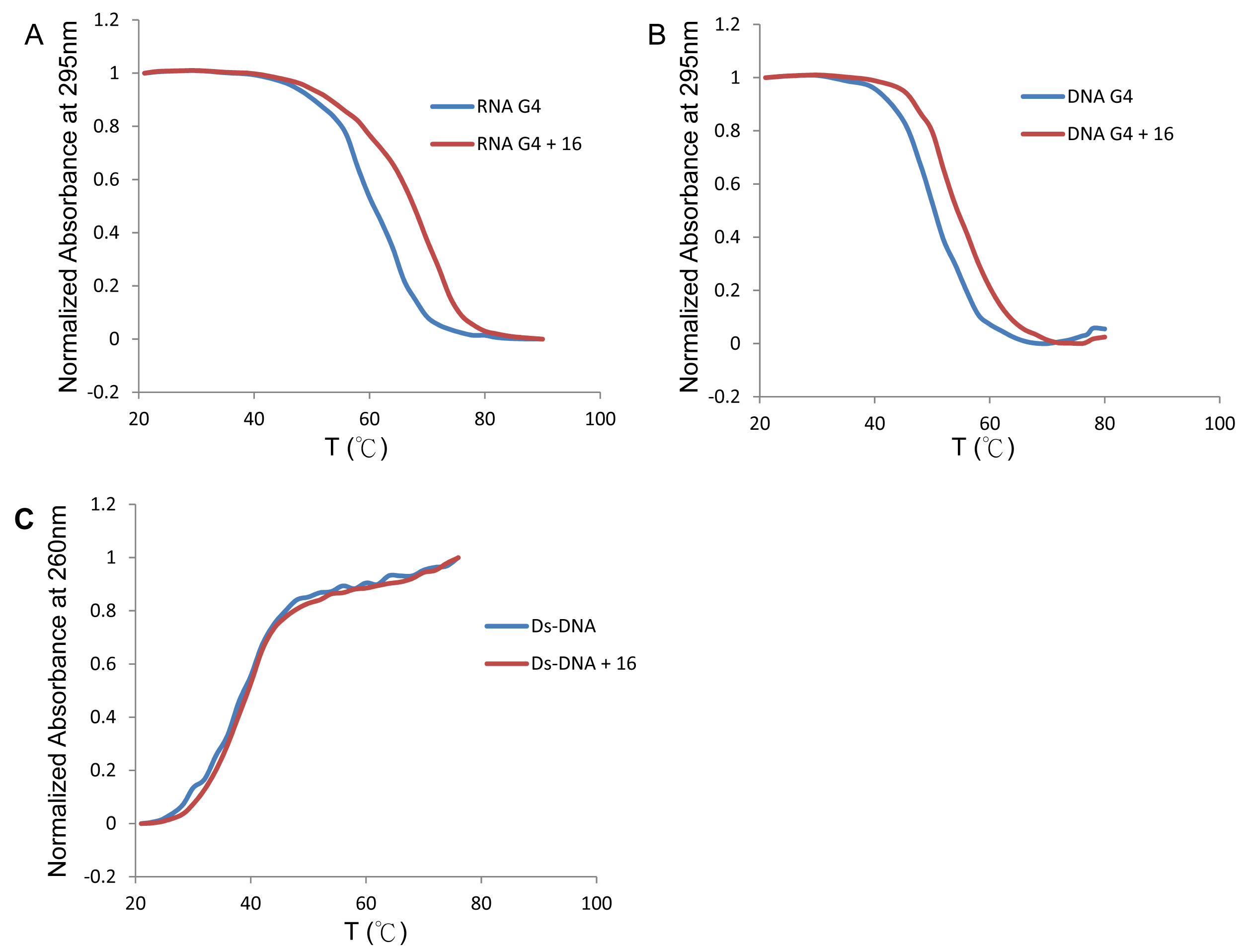

Supplement: Figure S13 — Thermal denaturation profiles of oligonucleotides in the absence and presence of 16. (A) UV-melting profiles of rAGGG(UUAGGG)3 (5 µM) in the absence and presence (50 µM) of compound 16 in 25 mM Tris-HCl buffer (pH 7.0) containing 5 mM KCl and 1% DMSO, (B) UV-melting profiles of dAGGG(TTAGGG)3 (5 µM) in the absence and presence (50 µM) of compound 16 in 25 mM Tris-HCl buffer (pH 7.0) containing 5 mM KCl and 1% DMSO, (C) UV-melting profiles of double-stranded oligodeoxynucleotide (Ds-DNA) 5′-AGGGTTAGGGT-3′/3′-TCCCAATCCCA-5′ (5 µM) in the absence and presence (50 µM) of compound 16 in 25 mM Tris-HCl buffer (pH 7.0) containing 1% DMSO. (TIF) [file pone.0053962.s013.tif]
